# Can application and transfer of strategy be observed in low visibility condition?

**DOI:** 10.1371/journal.pone.0173679

**Published:** 2017-03-13

**Authors:** Imen El Karoui, Kalliopi Christoforidis, Lionel Naccache

**Affiliations:** 1 Inserm U1127, Paris, France; 2 CNRS, UMR 7225, Paris, France; 3 Sorbonne Universités, UPMC Univ Paris 06, UMRS 1127, Paris, France; 4 Institut du Cerveau et de la Moelle épinière, ICM, Paris, France; 5 AP-HP, Hôpital de la Pitié Salpétrière, Department of Neurophysiology, Paris, France; University of Cambridge, UNITED KINGDOM

## Abstract

It has been long assumed that cognitive control processes can only be applied on consciously visible stimuli, but empirical evidence is contradictory. In the present study, we investigated strategic adaptation to conflict both in unmasked and in low-visibility masked trials. Using a paradigm derived from the Stroop task, we studied the application of strategies, but also the transfer of a strategy developed in unmasked trials to masked trials, and the trial-to-trial dynamics of strategic processing. In unmasked trials, we found evidence of strategic adaptation to conflict, both in reaction times and in ERPs (N2 and P300). In masked trials we found no evidence of behavioral adaptation to conflict, but a modulation of the P300 was present in masked trials included in unmasked blocks, suggesting the existence of a transfer of strategy. Finally, trial-to-trial analyses in unmasked trials revealed a pattern suggestive of dynamic subjective adherence to the instructed strategy.

## Introduction

Cognitive control refers to our ability to rapidly and flexibly adapt our behavior depending on our current goals and environment. Several paradigms have been designed to study this process, including task switching paradigms in which subjects have to respond to the same stimuli in different ways according to the context [1], or stop-signal tasks in which a stimulus requires the participant to stop his action [2]. Cognitive control can also be observed in interference tasks. A classic example of such a task is the Stroop task [3]. In this paradigm, subjects are presented with color words (e.g. red) printed in a colored ink and they have to respond to the ink color, independently of the word meaning. The ink color and the color associated with the meaning of the word can either be congruent (“red” printed in red) or incongruent (“red” printed in green). In incongruent trials, there is a response conflict between the relevant information (the color of the ink) and the irrelevant information (the meaning of the word), and participants are thus typically slower to respond than in congruent trials. This effect is known as the Stroop effect. Interestingly, it can be modulated depending on the context: the influence of irrelevant information is reduced when response conflict is frequent (e.g. in a block of trial—[4]) or recent (e.g. in the previous trial—[5]). This Gratton effect reflects the adaptation to response conflict and is a typical example of cognitive control.

A debated issue is whether cognitive control can be triggered unconsciously (for reviews see [6–9]). Indeed, cognitive control has been proposed to be only reserved for consciousness [10,11]. But recent experimental evidence challenges this view. For example, van Gaal et al. [12] showed that participants slowed down their responses following an unconscious stop signal, suggesting that it was partially processed but not enough to fully inhibit their response. However, experimental evidence for unconscious cognitive control is ambiguous: triggering cognitive control by explicit events such as a stop signal or a task-switching signal [13] seems to be possible even if this signal is unconscious, but when the event triggering cognitive control is derived from regularities in the environment, such as the frequency of response conflict, the results are much more disputed (for review see [8]). Note that explicit events such as stop signals have to be learned in conditions in which they are clearly visible, before they can be used in masked trials. Conflict adaptation on a trial-to-trial basis when the stimulus triggering the conflict itself is masked has been studied for example by Kunde [14]. In this study using meta-contrast masking, adaptation to conflict was observed only after a clearly visible trial, which suggests that a masked conflict cannot alter the processing of the next trial. However, van Gaal et al. [15] used a similar paradigm, shortening the inter-trial interval and omitting a warning sound at the beginning of each trial. They observed conflict adaptation on the current trial, independently of the visibility of the previous trial. This study thus suggests that trial-to-trial conflict adaptation could be triggered by a masked conflict (see also [16,17]), but it can also be interpreted alternatively as the consequence of the awareness of a meta-cognitive information, such as reaction time slowing [18]. Another interesting example is the work of Merikle and colleagues. They showed in a series of studies that subjects used predictive strategies based on blockwise conflict frequency only when the conflict was clearly visible [19–21]. These studies were based on a variant of the Stroop task: subjects were presented with a color prime word (red or green in grey color) for 33 ms that was followed by a target which consisted of seven colored ampersands, and they were asked to respond to the color of this target as quickly as possible. A pattern mask consisting of seven ampersands in gray color was presented between the prime and the target, either immediately after the prime in the low visibility condition or after a delay of 134 ms in the high visibility condition. The stimulus onset asynchrony (SOA) between the prime and the target was held constant across high and low visibility trials (300 ms). The prime and the target were incongruent in 75% of the trials. The authors found an interaction between the visibility of the prime and the prime-target conflict: subjects responded faster on incongruent than on congruent trials in the high visibility condition (reverse Stroop effect), whereas they responded slower on incongruent than on congruent trials in the low visibility condition (Stroop effect). These studies suggest that strategic blockwise adaptation to conflict can only be observed for clearly visible stimuli and were later replicated [22]. However, these results have been challenged by several studies showing blockwise adaptation to masked conflict frequency manipulations [17,23–25]. Moreover, it has been shown that context-specific blockwise conflict adaptation can be observed independently of conflict visibility and context identification, although it depends on the temporal proximity of context and conflict information [26].

In addition to behavioral measures, it is possible to study conflict processes using various measures of neural activity [27], and in particular in relation to frontal lobe activity. Indeed, the activity in the anterior cingulate cortex (ACC) has been associated with response conflict monitoring using functional MRI (fMRI) and Positron Emission Tomography (PET) [28–32]. The dorsolateral prefrontal cortex (DLPFC) and the supplementary motor area (SMA) have also been associated with conflict adaptation [32–34]. In addition to these studies providing a good spatial resolution, electroencephalography (EEG) was used to better understand the time course of conflict adaptation. Two event-related potentials (ERPs) were identified. The first one is the fronto-central N2 component peaking between 200 ms and 350 ms after stimulus onset, which, according to source reconstruction studies, is generated in the ACC [35–37]. In agreement with the role of ACC in the conflict monitoring theory [31], the amplitude of this component is higher on incongruent compared to congruent trials [38–40]. The second component modulated by congruence is the centro-parietal P300, peaking 300 to 500 ms after stimulus onset [38,41,42]. Its functional significance is less clear than the N2 components. It has been associated to response inhibition [41] or response evaluation [43].

In the present study, using both behavioral and EEG data, we aimed at exploring in more detail blockwise and trial-to-trial adaptation to conflict, how they interact and how they relate to conflict visibility, with an experimental paradigm similar to the one used by Merikle and colleagues’ studies [19–21]. Importantly, we introduced two baseline conditions (masked and unmasked) in order to detect any change in the Stroop effect relative to the high proportion of incongruent trials. Indeed in previous studies, only a reversal of the Stroop effect was considered as reflecting strategic processing [19,20,22,44], whereas the comparison with a condition with 50% incongruent could reveal more subtle effects. Moreover, we explicitly instructed subjects about the high proportion of incongruent trials [22], in order to test the mere application of the strategy that subjects did not have to infer. We also decided to use a short stimulus-onset asynchrony (SOA) between the prime and the target, compared to previous studies in which no strategic effect was found in masked trials [19,20,22,44], as subliminal priming is known for decreasing with time [45,46]. Note also that in the studies showing blockwise adaptation to conflict in masked trials, a short SOA between the prime and the target was used [23–25,47]. Finally, we added a new experimental condition for masked trials by comparing blocks exclusively composed of masked trials with blocks including a minority of masked trials randomly intermixed with unmasked trials. We speculated that even if no strategic processing was observed in fully masked blocks,—as reported by Merikle and colleagues’ studies [19–21] -, the strategy deployed on unmasked trials may well transfer to masked trials [48]. Indeed several recent studies demonstrated that far from being necessarily automatic and immune to top down control, processing of masked stimuli can be modulated by various factors such as endogenous spatial and temporal attention [49,50], task instructions or the current conscious semantic context [51]. This means that we may be able to observe a transfer of the current conscious strategy, elaborated during high proportion of unmasked incongruent trials, to masked trials embedded in unmasked blocks. Such a finding would thus enlarge the growing collection of evidence suggesting that conscious processing influences on masked trials. These top-down influences on the processing of masked stimuli are crucial regarding theoretical issues, and they could also be important for everyday life concerns such as ergonomic questions [52] or optimization of our attention resources to relevant information.

Using this adapted paradigm, we first aimed at replicating the strategic blockwise adaptation to conflict in unmasked trials and we analyzed how the trial-to-trial dynamics of conflict adaptation can be related to the blockwise strategy. Second, we studied blockwise adaptation to conflict in masked trials with explicit instructions and tested the mere application of an instruction in masked blocks. Finally, we tested the hypothesis that once established on unmasked trials, the strategic use of the prime could be transferred to masked trials, by including 30% masked trials in unmasked mostly-incongruent blocks.

## Materials and methods

### Subjects

Twenty-one right-handed subjects (10 women, age mean = 23.2 years, sd = 2.4) were included in this study. They all reported normal or corrected-to-normal visual acuity. The experiment was approved by the ethical committee of the Pitié-Salpétrière hospital. All subjects gave their written informed consent and were paid 40€ to participate in the experiment.

### Stimuli and procedure

**Fig 1:**
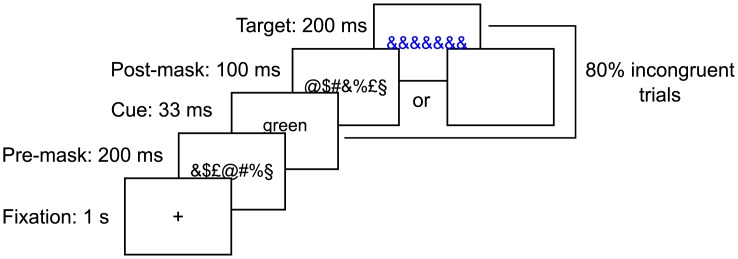
Experimental paradigm. We used a modified version of the Stroop task. Subjects were first presented with a color word as a prime (blue or green) and they had to respond to the color of a meaningless string. The prime could either be masked or unmasked. There were 4 types of blocks: masked baseline (50% incongruent trials), unmasked baseline (50% incongruent trials), mostly-incongruent masked blocks (80% incongruent trials) and unmasked mostly-incongruent blocks (80% incongruent trials) which included 70% unmasked trials and 30% masked trials.

Stimuli were presented against a gray background at the center of a 17-inch LCD Dell screen (frequency 60 Hz, resolution 1290 x 1024). We used a modified version of the Stroop task. Each trial started by a fixation cross that lasted 1s, then a pre-mask composed of the characters &$£@#%§ ordered randomly was presented for 200 ms (Fig 1). A prime consisting of a color word (green or blue) was then presented for 33 ms and was followed for 100 ms by either a post-mask composed of the same characters as the pre-mask but in another random order in masked trials, or by a blank screen in unmasked trials (stimulus-onset asynchrony (SOA) = 133 ms). Finally, a target consisting in a blue or green string of ampersands (&&&&&&) was presented for 200ms and subjects had to respond to the color of this string by pressing a button with their left or right hand. The color/hand correspondence was counterbalanced across subjects. After subject response (we waited for their response up to a limit of 10 seconds), the next trial sequence started following the same procedure. Therefore the inter-trial interval ranged from 1860±270ms. A pause was proposed after the completion of each block (pause duration = 77.9sec±41.8).

The prime and the target were either congruent (the word corresponded to the color of the target) or incongruent (the word did not correspond to the color of the target). Subjects were presented with 10 critical blocks of 100 trials containing a high proportion of incongruent trials (80%—mostly-incongruent condition). They were told about this high proportion of incongruent trials and were instructed to use the prime word to prepare the response corresponding to the opposite color, as this was the best strategy to answer rapidly and accurately in most trials. Among these 10 blocks, 2 blocks contained only masked trials and the 8 others contained 70% of unmasked trials and 30% of masked trials. These blocks allowed us to test the development of the strategy on fully masked blocks but also on masked trials embedded in unmasked blocks, in which the strategy established on unmasked trials could be transferred to masked ones. The two blocks containing only masked trials were presented either before or after the 8 other blocks. Their position was randomized across subjects. Finally, subjects were presented with 2 blocks of 100 unmasked trials with 50% incongruent trials (unmasked baseline condition) and 2 blocks of 100 masked trials with 50% incongruent trials (masked baseline condition). These blocks of baseline were presented at the very beginning (one block of each) and at the end of the experiment (one block of each). The order of the unmasked and masked baseline was random. Feedback on reaction time and accuracy was provided to subjects at the end of each block. In total, subjects performed 1400 trials.

At the very end of the experiment, subjects performed a discrimination task on the prime, in 2 distinct blocks of 100 trials: the first one comprised only masked trials and the second one comprised 70% of unmasked trials and 30% of masked trials, as in the main task. In these blocks, 50% of trials were congruent. Their order of presentation was counterbalanced across subjects. The precise procedure of stimulus sequence followed exactly the same parameters as in the main experiment. Finally, subjects provided informal subjective reports about the visibility of the prime during the two discrimination blocks. In total, the experiment lasted about 45 min.

### Behavioral data analysis

Incorrect trials and correct trials with reaction times faster than 200 ms or slower than 800 ms (representing 2.3% of trials) were excluded from all analyses on reaction times. Moreover, when studying trial-to-trial conflict adaptation, the first trial of each block and trials following an error were excluded. For error rate analyses, all trials were included. Behavioral data were analyzed with MATLAB R2011a (The MathWorks, Inc.) and the Statistical toolbox to perform t-tests and repeated-measures ANOVA when comparing the different conditions. The normality assumption required by these parametric tests was checked with a chi-square goodness of fit test. It was met for all conditions (all χ^2^ values < 0.42, all p < 0.05).One subject was excluded due to excessive artefacts on the EEG signal. So the results presented are based on 20 subjects. For each subject, we averaged all trials of a given condition, and then computed the ANOVAs on the distribution of mean values across subjects and conditions (see S1 File and S1 Table for details).

### EEG recordings and analyses

EEG activity was continuously recorded using a 256-channel EEG net (Electrical Geodesics Inc, Eugene, OR). The signal was sampled at 250 Hz.

All analyses were conducted using Fieldtrip toolbox [53]. EEG data were filtered between 0.5 and 30 Hz with a two-pass Butterworth filter and then epoched from -400 ms to 900 ms relative to the onset of the target. Then, the data were visually inspected for artefacts not related to blinks. Bad channels were interpolated with the weighted average of all their neighbors. Independent components analysis was computed and components containing blinks or oculomotor artefacts clearly dissociable from brain activity were subtracted from the data. Finally, the data were re-referenced to common average and baseline corrected over 200 ms before the prime, between -400 and -200 ms relative to the onset of the target.

To improve signal-to-noise ratio and based on previous literature [35,41,54,55] about the components of interest (N2 and P3), a spatial region-of-interest (ROI) including 15 channels was created around Cz. Note that an EEG system with lower spatial sampling could have been used to perform this study, as we focus only on a ROI.

To assess the significance of differences between congruent and incongruent trials in the different conditions, we used dependent sample t-tests across subjects at each time point in the ROI around Cz. Then correction for multiple comparisons over time was performed, by nonparametric cluster-based method [56]. We computed N = 1000 permutations by shuffling trial labels. Then, for each permutation, dependent sample t-tests were performed at each time sample. All samples with a t-value corresponding to a p-value smaller than 0.05 were clustered in connected sets on the basis of adjacency in time. Then the cluster statistic was computed by taking the sum of the t-values within each cluster. The cluster-corrected threshold was obtained by computing the permutation distribution of the maximum cluster statistic and taking the *c+1* largest member of this distribution, with *c* is equal to αN rounded down. In the original data, only clusters with a cluster statistic higher than this threshold were identified as significant. All the reported p-values are corrected for multiple comparisons.

## Results

### Behavior

Adaptation to conflict frequency was studied in 5 conditions: unmasked and masked baseline conditions (with 50% incongruent trials), unmasked and masked mostly-incongruent conditions (with 80% incongruent trials), as well as masked trials included in unmasked mostly-incongruent blocks. We analyzed the Stroop effect, as measured by the difference in reaction times between incongruent and congruent trials. Note that this effect was not significantly different between the baseline block included at the beginning of the experiment and the baseline block presented at the end of the experiment, both for masked (t(19) = -1.57, p = 0.13) and unmasked (t(19) = 1.03, p = 0.32) conditions. We thus collapsed both blocks for each baseline condition, in order to increase statistical power.

#### Assessment of prime visibility

Visibility of the masked prime was analyzed through d’ scores on the discrimination task and subjective reports at the end of the experiment, according to which participants were not able to identify the primes. In the block containing only masked primes, d’ scores were significantly different from 0 (mean = 0.28, sd = 0.33; t-test, t(19) = 3.78, p = 0.001), but they were not correlated with the Stroop effect in the masked baseline condition (Pearson correlation, r = -0.16, p = 0.48) and the intercept was significantly different from 0 (estimate = 0.31; t(19) = 3.6, p = 0.002). In the block containing only 30% of masked trials, d’ scores were significantly different from 0 (mean = 1.14, sd = 0.64; t-test, t(19) = 7.95, p < 0.001), but they were not correlated (r = 0.14, p = 0.53) with the Stroop effect for the masked trials included in unmasked blocks (mean Stroop effect = 8.6 ms; t(19) = 2.3, p = 0.03) and the intercept was significantly different from 0 (estimate = 1.09; t(19) = 6.62, p <0.001). These results suggest that even if objective prime visibility (as indexed by d’ values) was above chance-level, it could not easily explain the conflict effect that was not correlated with it, and that was interpolated as still significant for a theoretically null d’.

#### Strategic blockwise adaptation to conflict

The unmasked baseline condition was used to assess the existence of the Stroop effect in the unmasked condition (mean Stroop effect = 23 ms; t(19) = 4.57, p < 0.001). Then the masked baseline condition was analyzed to test that the masked prime was correctly processed. There was a significant Stroop effect in the masked baseline condition (50% incongruent trials) (mean = 7.3 ms; t(19) = 2.32, p = 0.03).

**Fig 2:**
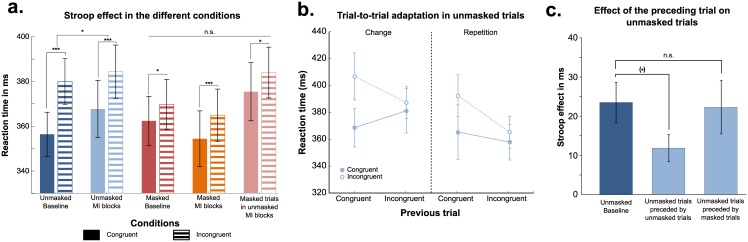
Behavioral results. (a) Stroop effect, as measured by the difference in reaction times between incongruent and congruent trials, is presented in the different experimental conditions. The Stroop effect is strongly reduced in the unmasked mostly-incongruent (MI) blocks compared to baseline, but no difference relative to baseline is observed for masked trials. (b) Reaction time is presented according to the current trial congruence and the previous trial congruence, both for physical repetition and change across the previous and current trials. A significant trial-to-trial conflict adaptation effect was observed, that was not modulated by physical repetition factor. (c) Stroop effect for unmasked trials is presented according to the visibility and the congruence of the previous trial. It is significantly different from baseline only when the preceding trial is unmasked and incongruent, suggesting an online evaluation of the instructed strategy.

We performed a repeated-measures ANOVA on median RTs of correct trials with visibility (masked/unmasked), conflict (congruent/incongruent), incongruent proportion (baseline/mostly incongruent) declared as within-subjects factors, and subjects as a random factor (Fig 2a). We observed a main effect of conflict (Stroop effect—F(1,19) = 35.6; p<0.0001), as well as a main effect of visibility (F(1,19) = 4.9; p = 0.03). No main effect of incongruent proportion was present (F(1,19) = 0.01). A significant interaction was found between visibility and conflict (F(1,19) = 9.6; p = 0.006) reflecting a stronger Stroop effect for unmasked trials than for masked ones. Finally, a significant triple interaction was observed between visibility, conflict and incongruent proportion (F(1,19) = 5.5; p = 0.03). Post-hoc tests explained this triple interaction by a trend towards a decrease of the unmasked Stroop effect for mostly incongruent trials (mean Stroop effect = 16.7 ms) as compared to baseline ones (mean Stroop effect = 23.5 ms—unilateral p-value = 0.1 in the conflict X incongruent proportion interaction restricted to unmasked trials; Fig 2a), while no such a modulation was detected for masked trials (mean Stroop effect for mostly incongruent masked trials = 10.5 ms; mean Stroop effect for baseline masked trials = 7.3 ms—paired t-test: p = 0.4; Fig 2a). The same analysis on error rates showed no significant effect (all F<1).

In other terms, behavioral data suggested that a trend of a modulation of the Stroop effect by the proportion of incongruent trials was present exclusively for highly visible trials.

#### Trial-to-trial conflict adaptation

We then analyzed trial-to-trial effects in mostly incongruent blocks in order to better probe potential adaptive effects to dynamics of conflict at a finer time-scale. We report here results on unmasked trials, as no evidence of trial-to-trial adaptation was observed in masked trials (all Fs < 1). We analyzed the influence of the previous trial congruence on the Stroop effect [5] using only correct trials preceded by correct trials. First, we studied this influence in unmasked trials preceded by an unmasked trial. To take into account the role of stimulus repetition in this trial-to-trial conflict adaptation [57], we performed a repeated-measures ANOVA including current trial congruence (congruent/incongruent) and previous trial congruence (congruent/incongruent) as within-subject factors and subjects as a random factor (Fig 2b). We found a main effect of current trial congruence (F(1,19) = 33.3, p < 0.001) and a main effect of previous trial congruence (F(1,19) = 8.6, p = 0.01; with faster RTs for previously incongruent as compared to previously congruent trials). We found a trend (but in the predicted direction) of an interaction between these two factors (F(1,19) = 4.03, unilateral p = 0.03). Note that this interaction corresponds to the trial-to-trial Gratton’s adaptation effect: current trial conflict effect tends to me smaller after an incongruent trial than after a congruent one. No other effect was found significant. Note also that the same ANOVA including the physical repetition factor showed the absence of triple interaction between current trial congruence, previous trial congruence and physical repetition was not significant (F(1,19) = 0.57, p = 0.46), indicating that the absence of difference of trial-to-trial patterns for physical repetitions and changes.

Then, we aimed at better understanding the difference reported above between unmasked and masked trials: while a Stroop effect was observed in both conditions (baseline blocks), a strategic modulation of this effect during MI blocks was present exclusively for unmasked trials. We reasoned that if subjects could infer a relevant strategy in unmasked trials of MI blocks only, then we may be able to track the dynamics of the ‘subjective confidence’ in the relevance of this strategy. We predicted that strategy relevance should be increased when the preceding trial was both clearly visible (unmasked) and incongruent (matching the MI trials predictive strategy). We first confirmed that when the preceding trial was masked, its congruity (congruent/incongruent trial) did not impact current unmasked trial RTs (F(1,19) = 0.4; p = 0.5), and did not interact with current trial conflict effect (F(1,19) = 0.87; p = 0.4; no Gratton effect from masked to unmasked trials). Then we could also confirm the second condition of our prediction: conflict effect of MI blocks unmasked trials was indeed different than the one observed in unmasked baseline blocks exclusively after an unmasked incongruent effect (t(19) = 2.53, p = 0.02), while it did not differ from baseline conflict effect after a congruent trial disconfirming strategy relevance (t(19) = -0.99, p = 0.33).

In summary, we found a significant effect in the masked and unmasked baseline conditions, indicating that the prime was properly processed in both cases. A trend of strategic blockwise adaptation was only found for unmasked trials, and no evidence of application or transfer of strategy could be shown for masked trials. Moreover, we discovered that a dynamic adaption of MI block strategy: conflict effect in the MI block differed from the one measured in baseline blocks only when the preceding trial confirmed the strategy validity (incongruent trial) and was clearly visible. This effect is suggestive of a conscious strategic processing: subjects may have evaluated on a trial-to-trial basis the validity of their strategy according to consciously available information.

### Event-related potentials

**Fig 3:**
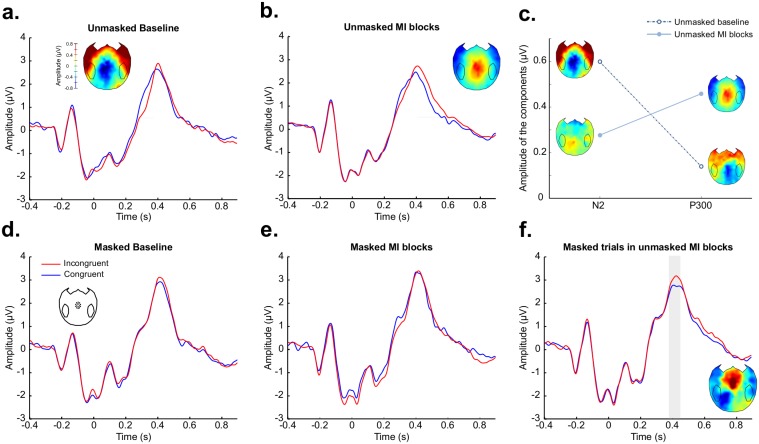
Two ERP components: N2 and P300. In panels (a), (b), (d), (e) and (f), ERP analyses from a cluster of 15 electrodes around Cz in different experimental conditions are presented, with incongruent trials in red and congruent trials in blue. The gray shadings indicate time periods of statistical significance (pcorrected < 0.05) and scalp topographies of the difference between incongruent and congruent trials show the effects of congruence during these time periods. (a) In the unmasked baseline, the N2 component is modulated by congruence from 272 ms to 364 ms after the target onset. (b) In unmasked mostly-incongruent (MI) blocks, the P300 component is modulated by congruence from 404 ms to 572 ms after the target onset. (c) The amplitude of the N2 and P300 components was computed as the absolute value of the difference between congruent and incongruent trials in the time windows of interest for each component. We found a significant interaction (F(1,19) = 6.67, p = 0.02) between the amplitude of the components and the experimental condition (unmasked baseline—dotted line/unmasked mostly-incongruent blocks—continuous line). Scalp topographies of the difference between incongruent and congruent trials for the time window of interest for each component and in each condition are plotted next to the corresponding point in the plot. (d) No significant difference was observed in the EEG between congruent and incongruent trials in the masked baseline condition. (e) No significant difference was observed in the EEG between congruent and incongruent trials in the masked baseline condition. (f) Interestingly, in the masked trials included in the unmasked mostly-incongruent blocks, a modulation of the P300 by congruence was observed from 384 ms to 452 ms after the target onset, similar to the signature observed for unmasked trials in mostly-incongruent blocks.

EEG activity was analyzed in the different conditions around Cz (Fig 3). Two main event-related potential (ERP) components modulated by prime-target conflict were identified. An N2 component significantly modulated by conflict (p_corrected_ = 0.007) was observed in the unmasked baseline condition from 272 ms to 364 ms after the target onset (peak difference = -0.84 peak latency = 320 ms–Fig 3a). A P300 component significantly modulated by conflict (p_corrected_ = 0.001) was observed from 404 ms to 572 ms after the target onset in the mostly-incongruent unmasked condition (peak difference = 0.61, peak latency = 480 ms–Fig 3b). We performed a repeated-measures ANOVA on the amplitude of these components with component (N2/P300) and condition (baseline/mostly-incongruent) as within-subject factors and subjects as a random factor. Interestingly, the interaction between these factors was significant (F(1,19) = 6.67, p = 0.02 –Fig 3c). Post-hoc tests explained this interaction by the fact that the amplitude of the P300 component is significantly higher in the mostly incongruent condition (mean amplitude = 0.46) than in the baseline condition (mean amplitude = 0.14 –paired t-test p = 0.03), whereas the amplitude of the N2 component tends to be higher in the baseline condition (mean amplitude = 0.60) than in the mostly incongruent condition (mean amplitude = 0.28 –paired t-test p = 0.06). These results suggest that the behavioral strategic processing specific to unmasked trials of MI blocks (see above) requires two distinct neural processes. The implementation of the strategy would be associated with the late P300 signature (“prepare a response opposite to the one associated with the prime”), while the inhibition of the automatic response elicited by incongruent trials would appear as a decrease of the N2 response.

For masked trials, no significant difference between congruent and incongruent trials was observed in the baseline and in the masked mostly-incongruent blocks (Fig 3d and 3e). But interestingly, a P300 significantly modulated by congruence (p_corrected_ = 0.02) was observed for masked trials in mostly-incongruent unmasked blocks from 384 ms to 452 ms (peak difference: 0.45, peak latency: 424 ms—Fig 3f). This modulation,—very similar to the effect observed for unmasked trials -, suggests the existence of a transfer of the strategy in these trials, even if this effect could not be observed in the behavior.

Finally, we aimed at probing the ERP result supporting the behavioral evidence of trial-to-trial evaluation of strategy validity in unmasked trials in the mostly-incongruent condition. We analyzed the ERP around Cz taking into account the congruence and visibility of the previous trial. Interestingly, we found a significant modulation of the P300 component by the congruence of the current trial only when the previous trial was unmasked and incongruent (i.e. when the previous trial supported the instructed strategy), both when the current trial was unmasked (t(19) = 3.64, p = 0.001) and when it was masked (t(19) = 2.1, p = 0.04). This last result can be interpreted as an ERP effect reflecting the level of conscious adherence to the strategy used in the MI blocks: when present this P300 modulation would reveal subjects anticipation to expect a target incongruent with the prime, whereas its absence would reflect the absence of such a strategic processing. Therefore, its absence after masked trials and after unmasked congruent trials may reflect the lack of adherence to this strategic mode when consciously available information is either too weak to reinforce the relevance of applying the strategy (masked trials), or in opposition to the strategy (unmasked congruent trials).

## Discussion

In the present study, we explored the existence of strategic adaptation to conflict for masked and unmasked trials in mostly incongruent blocks, using an experimental paradigm adapted from the Stroop task. We observed a trend of blockwise adaptation to conflict in unmasked trials in behavioral data, and could confirm, strengthen and better explain its mechanism in EEG data. However, despite behavioral evidence of processing of masked stimuli, blockwise adaptation to conflict was not observed in these trials. This negative result was found both when masked trials were included in fully masked blocks and when they were embedded within mostly unmasked blocks. Thus, no evidence for application or transfer of the strategic use of the masked prime was found in the behavior. Nevertheless, in the ERP analysis, the modulation of the P300 component by prime-target congruence observed in ‘mostly incongruent’ blocks for unmasked trials was also present for masked trials embedded in unmasked blocks. This last finding suggests the existence of a transfer of strategy from unmasked trials to masked trials. Finally, detailed analyses of trial-to-trial adaptation to conflict in unmasked trials revealed original effects related to the visibility and the congruence of the previous trial.

### Processing of unmasked trials

In unmasked trials, as Merikle and colleagues [19,20], we found a trend of an impact of the strategy on the Stroop effect in the mostly incongruent condition. Note however that in our case the modulation of the Stroop effect compared to the baseline did not extend to a full inversion as reported by Merikle and colleagues. This difference can be explained by the short SOA we used (133 ms), in order to keep masked and unmasked trials as similar as possible and to maximize processing of masked stimuli. However, it has been shown that the SOA between the prime and the target should be at least 300 ms to observe a reversal of congruence effect [19,20,22]. Nevertheless, including a baseline condition allowed us to show the existence of strategic processing in unmasked trials, even with a short SOA. However, it should be noted that this difference between baseline and mostly-incongruent conditions could be interpreted as a simple inhibition of prime processing, rather than real strategic processing. Further studies should be conducted to dissociate these two possible interpretations.

Moreover, the ERP analysis in unmasked trials revealed that two components were modulated by the task. Indeed, we observed that the N2 was suppressed in mostly incongruent blocks compared to the baseline, whereas the P300 was modulated by congruence only in mostly incongruent blocks and not in the baseline condition. This interaction between the component (N2/P300) and the condition (mostly incongruent/baseline) suggests that the strategic adaptation to conflict impacts both early conflict detection processes as reflected by the decrease in the N2 component [35–37] and late strategic processes indexed by the P300 [41,42]. This finding is noteworthy as it highlights that even early processes can be affected by top-down strategic control. It is in the agreement with the conflict-monitoring theory [30,31], according to which the anterior cingulate cortex (ACC) activation related to conflict detection and indexed in EEG by the N2 [36] should be lower when incongruent trials are frequent [28,33]. Indeed, finding an interaction between the component (N2/P300) and the condition (mostly incongruent/baseline) means that N2 and P3 effects varied in opposite directions in relation to the level of Stroop conflict: when the N2 effect decreased from baseline blocks (with 50% incongruent trials) to high-conflict blocks (80% incongruent trials in mostly-incongruent blocks), the P3 effect increased from baseline to high-conflict blocks. Given that in the present dataset, N2 and P3 components occur respectively earlier and later than the mean RTs, this pattern fits nicely with the standard interpretation of the inter-trial adaptation effect (Gratton effect). During the baseline blocks in which congruent and incongruent trials are equally likely and not predictable, the processing of a conflicting trial (incongruent) induces a high level of internal conflict reflected in the amplitude of the N2 component. Then after the processing of this conflicting stimulus, control systems increase the level of cognitive control for the next trial to come, as evidenced by the P3 component. This trial-to-trial regulation would explain why immediately after a conflicting trial, the increase of cognitive control causes a reduction of internal conflict induced by an incongruent trial (N2) and a decrease in the behavioral Stoop effect. In mostly incongruent blocks during which we observed a strong reduction of the Stroop effect, this strategical effect is expected to be even more relevant and efficient than in baseline blocks. Therefore, our dual observation of a reduction of N2 and increase of P3 when comparing baseline and mostly incongruent blocks is in line with the expected pattern of strategical processing of primes: a high level of control (P3) enables a net reduction of internal conflict (N2).

### Masked prime visibility

Visibility of masked primes was assessed by combining subjective reports and an objective forced-choice discrimination task in two distinct conditions which mimic the trial structure used in the main experiment: one block contained only masked trials whereas the second contained 70% unmasked trials and 30% masked trials. All volunteers denied having perceived consciously any prime stimulus, neither during the main experiment nor after the two discrimination blocks. Nevertheless, objective measures (d’) of direct prime processing were significantly better than chance-level in both discrimination blocks. This pattern of results prevents any strong claim about the absence of conscious perception of masked primes, even if a positive d’ does not constitute a univocal evidence of conscious perception [58]. This constitutes an obvious limitation of the current study which could have been overcome by collecting subjective reports of conscious visibility of primes on a single-trial basis [59]. In order to probe the fine dynamics of inter-trial processing, we decided not to interrupt subjects’ engagement in the main task by collecting such single-trial subjective reports. Note that this limitation reinforces in a way our behavioral findings concerning these masked trials. Indeed, if no evidence of behavioral adaptation to conflict was detected for these masked trials for which we cannot guarantee the absence of conscious perception, all the more so for invisible stimuli (with null d’ values on objective discrimination tasks). Interestingly, the d’ scores obtained in the block with both masked and unmasked trials were higher than those obtained in the block with only masked trials. This result could reflect either an increased conscious visibility or an increased unconscious objective performance and is reminiscent of previous studies on the influence of temporal and spatial attention and stimulus expectations on processing of masked stimuli [49,50,60–63] and on improved conscious visibility of stimuli presented at threshold [64]. Indeed, in the block with both masked and unmasked trials, subjects had strong expectations about the masked stimuli, which probably facilitated their perception. In the present study, we did not collect detailed subjective visibility assessment for each trial, so we cannot disentangle between the hypothesis of an increased conscious visibility and the hypothesis of an increased unconscious objective performance. Note, however, that we did not observe evidence of behavioral adaptation in masked trials, despite high d’ scores, suggesting a different processing in these low visibility trials. Moreover, the absence of richer subjective report for high-d’ blocks (masked trials within unmasked trials) than for low-d’ blocks (masked only trials) suggest that this d’ performance may reflect a stronger unconscious perception than an increase in conscious visibility of primes. Another possible explanation of these results could be that detecting the masked stimuli in the block with only masked trials is much more difficult and leads to a decrease in motivation as the task do not seem to make any sense as the subjects report seeing nothing [65].

In contrast with these negative behavioral results, our positive ERP finding of a P300 modulation for masked trials embedded within unmasked trials requires replication under conditions of full invisibility of primes, in order to interpret it as an unconscious effect.

### Processing of masked primes

The presence of a classical behavioral conflict effect for masked stimuli demonstrates that these stimuli were processed, but we could not observe either trial-to-trial or blockwise adaptation effects to conflict in masked trials

Concerning the lack of trial-to-trial adaptation, one may relate it to the rather long inter-trial interval we used (1860±270ms). Indeed, as noted in the Introduction section, the most crucial distinction between the study by Van Gaal et al. [15] who reported a Gratton effect in masked trials, and the negative finding of Kunde [14] relates to the inter-trial interval value.

Concerning the lack of blockwise adaptation effect in the present study, it is valuable to compare this negative finding to the observation of Jáskowski et al. [23] who showed such an effect in masked trials. They explained their results by a meta-cognitive process: they assumed that subjects were aware of the consequences of the high proportion of incongruent trials (higher number of errors) and regulated their behavior accordingly, even though they were not aware of the conflict itself. This interpretation fits with the idea that masked stimuli cannot trigger cognitive control directly, but that the reportable consequences of these stimuli can [11,66,67]. Note that the priming effects in Jáskowski et al.’s study are very strong (about 100 ms difference in reaction times and about 15% error rate in incongruent trials), allowing subjects to notice changes in these parameters. The absence of a modulation in the masked condition in the present study might be explained in this meta-cognitive framework. In our experiment, the Stroop effect is small (e.g. for the masked baseline, mean = 8.8 ms) with low error rate for incongruent trials (e.g. for the masked baseline, mean = 5.6%), so it might be hypothesized that participants did not notice consciously the difference between congruent and incongruent masked trials and thus could not modulate their behavior in these trials. However, in our study, the instructions were explicit but no modulation according to the proportion of incongruent masked trials was observed, suggesting that knowing consciously the structure of the task is not sufficient for subjects to adapt their behavior in masked trials and that this information should be extracted from responses themselves. Note that, in our study, the modulation of the Stroop effect requires semantic processing of the prime, whereas the paradigm used by Jáskowski et al. requires only lower-level processes to identify the direction of an arrow, which could explain the observed differences in the amplitude of the priming effects [68,69]. Other interesting accounts, which do not rely on the notion of conflict, have been developed to explain the effects of the proportion of incongruent trials in a given block [70–72]: temporal learning and contingency learning. For example, the so-called adaptation to statistics of the environment (ASE) model highlights the importance of difficulty level of the previous trial to explain changes in reaction times in the current trial [70,71]. Further studies need to be performed to test the contribution of these different processes in blockwise adaptation to conflict.

Moreover, the analysis of the ERPs revealed a modulation of the P300 component by congruence in masked trials included in mostly unmasked blocks, but not in masked trials included in fully masked blocks. Interestingly, in unmasked trials, this modulation was observed in mostly incongruent blocks, but not in the baseline condition, suggesting that it reflects a process involved in the strategic use of the prime. Therefore the P300 modulation observed in masked trials included among unmasked trials could be explained in terms of cognitive control triggered by unmasked stimuli, and is consistent with previous studies [55]. Note that this effect was not present in the behavioral data, suggesting that EEG data can reveal some processes which cannot be detected in behavior [27,73]. This modulation of the P3 component may also reflect a metacognitive process enabling a conscious appraisal of high-level of internal conflict induced by low-visible stimuli. Replications of this experiment enriched by the collection of subjective reports about the current level of internal conflict would be necessary in order to test this hypothesis.

The second ERP finding which is surprising in the current study is that the modulation of the N2 component by congruence observed in the unmasked baseline is not present in the masked baseline, although the behavior shows a differential processing of congruent and incongruent trials in this condition. This result might be explained by the relatively low number of trials in the baseline condition as well as by the low amplitude of behavioral effects in masked trials. Note that in a previous study contrasting mostly-congruent and mostly-incongruent blocks, Jiang et al. found an N2 component in the strongly masked condition, which was modulated by block type [55]. The difference between this study and our current results could be explained by the difference in the experimental paradigm: Jiang and colleagues used arrows which trigger stronger congruence effects.

### Dynamics of strategical processing of unmasked trials

Finally, we found that the use of the instructed strategy depended on the previous trial congruence and visibility, suggesting an online evaluation of this rule from trial to trial. First, following an unmasked congruent trial, the Stroop effect was similar to the one observed in the baseline condition and the modulation of the P300 component by congruence, which indexed the application of the instructed strategy, was not present. Second, following a masked trial, independently of its congruence, the Stroop effect in unmasked trials was similar to the one observed in the baseline condition, and no P300 modulation by congruence was observed. Note that the modulation of the P300 observed in masked trials intermixed with unmasked trials (see above) do not seem to influence the processing of subsequent unmasked trials. This may be explained by the evanescence of masked effects [45,46] and related to the fairly long inter-trial interval used in the present study ([14,15]). Finally, we showed that all these results could not be explained by physical repetitions across trials [57]. The exact relation between trial-to-trial and blockwise conflict adaptation remains unresolved: they could either depend on the same mechanisms [71] or rely on different processes [74]. However, the trial-to-trial differences observed in the present study suggest that the instructed strategy is evaluated on each trial based on the recent history, as the Stroop effect was strongly reduced relative to baseline only after incongruent trials, which are valid with the strategy. Therefore, these differences may be described as the dynamics of ‘subjective strategy adherence’, which were observed exclusively for unmasked trials. This result may suggest that changes in subjective strategy adherence require conscious access to the stimuli, but this hypothesis requires additional studies in which forced choice discrimination performance (d’) on the masked stimuli will be closer to chance-level. An important issue would be to measure reportability of such strategy adherence, as this process could require conscious access to stimuli without being reportable itself. It would thus be interesting to test how much subjects adhere to the instructed strategy, by assessing their confidence in the strategy on each trial in future experiments. The modulation of strategy transfer to masked trials by the strength of conscious cognitive control processes as indexed by the trial-to-trial adherence to the strategy should also be tested.

## Supporting information

S1 File Additional information about the statistical method used for behavioral analysis. (DOCX)

S1 Table Number of trials for each experimental condition. (DOCX)
