# Supplementary material for: Can application and transfer of strategy be observed in low visibility condition?
Source: PLoS One. 2017 Mar 13;12(3):e0173679. doi: 10.1371/journal.pone.0173679 (PMC5348023; doi:10.1371/journal.pone.0173679)
Supplement: S1 File — (DOCX) [file pone.0173679.s001.docx]

**Supporting Information**

**S1 File. Additional information about the statistical method used for behavioral analysis**

For each subject, we averaged all trials of a given condition, and then computed the ANOVAs on the distribution of mean values across subjects and conditions. We did not control for trial numbers across conditions for several reasons. First, we preferred to keep all trials rather than selecting across multiple n-matching runs. Second, note that all our experimental conditions ranged from 40 trials (MI masked congruent trials) up to ~448 trials (see Table S1). Matching to the smallest number of trials did not appear to us as the single solution. Rather we examined the averages ERPs of all conditions and checked that no noisy effect was induced by this unequal number of trials. Note that the visual aspect of grand-average ERPs across all examined and plotted conditions, as well as our different statistical analyses did not call for n-matching procedure.
