# Supplementary material for: Can application and transfer of strategy be observed in low visibility condition?
Source: PLoS One. 2017 Mar 13;12(3):e0173679. doi: 10.1371/journal.pone.0173679 (PMC5348023; doi:10.1371/journal.pone.0173679)
Supplement: S1 Table — (DOCX) [file pone.0173679.s002.docx]

**Supporting Information**

**S1 Table. Number of trials for each experimental condition**

| Baseline | | Baseline | | MI | MI | MI | MI | M in UM | M in UM |
| --- | --- | --- | --- | --- | --- | --- | --- | --- | --- |
| UM | UM | UM | UM | UM | UM | M | M | M | M |
| Icg | Cg | Icg | Cg | Icg | Cg | Icg | Cg | Icg | Cg |
| 100 | 100 | 100 | 100 | 448 | 112 | 160 | 40 | 120 | 120 |
